# Factors Influencing Leaks in Primary Intestinal Anastomoses: An Observational Study

**DOI:** 10.7759/cureus.85133

**Published:** 2025-05-31

**Authors:** Vijaykharthik LK, Manivannan Rajarathinam

**Affiliations:** 1 General Surgery, Sree Balaji Medical College and Hospital, Chennai, IND

**Keywords:** anastomotic leak, body mass index (bmi), comorbidities (renal disease, diabetes, hypertension, intestinal anastomosis, risk factors, surgical complications

## Abstract

Background

An anastomotic leak (AL) remains a major complication in gastrointestinal surgery, particularly after primary intestinal anastomosis. It contributes to increased morbidity, prolonged hospitalization, and mortality.

Objective

To identify and evaluate patient, surgical, and perioperative factors associated with the development of anastomotic leaks in individuals undergoing a primary intestinal anastomosis.

Methods

This observational study was conducted at Sree Balaji Medical College and Hospital, Chennai, and included 70 patients who underwent primary intestinal anastomosis. Data were collected on demographics, comorbidities, surgical details, postoperative outcomes, and complications. Statistical analyses included logistic regression and receiver operating characteristic (ROC) analysis to assess predictive factors.

Results

The overall incidence of AL was 23.3% (14 out of 70 patients). Higher BMI (p = 0.005), renal disease (p = 0.043; 7 out of 9 patients with renal disease), hypertension (p = 0.041; 5 out of 6 patients), and diabetes (p = 0.029; 5 out of 7 diabetic patients) were significantly associated with AL. Logistic regression showed BMI to be an independent predictor of AL. An ROC analysis indicated predictive utility for BMI and CRP. No significant associations were found with age, gender, smoking, surgical duration, or blood loss.

Conclusion

BMI and comorbidities such as renal disease, hypertension, and diabetes are key risk factors for AL. Preoperative optimization and risk stratification based on these factors can improve surgical outcomes. Future studies should validate these findings in larger, multicentric populations.

## Introduction

A primary intestinal anastomosis is a widely practiced surgical technique aimed at restoring bowel continuity after resection, commonly for colorectal cancer, bowel obstruction, or traumatic injuries. Despite surgical advancements, anastomotic leak (AL) remains one of the most feared complications, with incidence rates ranging from 3% to 19% [1,2]. An anastomotic leak (AL) is associated with prolonged hospital stay, increased ICU admissions, high morbidity, and mortality [3].

The pathogenesis of AL is multifactorial. Patient-related factors such as obesity, diabetes, renal insufficiency, smoking, and poor nutritional status contribute significantly to impaired healing [4,5]. Similarly, surgical factors, including poor perfusion, tension at the anastomosis site, emergency procedures, and the surgeon’s technical proficiency, also play a role [6,7]. Literature shows that no single factor sufficiently predicts AL, necessitating a composite evaluation of risk. Recent innovations, such as fluorescence-guided imaging for perfusion and machine learning-based prediction models, have been explored to minimize the AL risk, but their clinical adoption is limited [8-10]. Hence, evaluating real-world patient and surgical variables can fill the evidence gap.

This study was undertaken to evaluate patient demographics, comorbidities, surgical techniques, and biochemical markers associated with anastomotic leaks in a tertiary care setting.

## Materials and methods

This prospective observational study was conducted in the Department of General Surgery at Sree Balaji Medical College and Hospital from January 2022 to June 2023 (18 months). A total of 70 patients undergoing primary intestinal anastomosis were recruited using purposive sampling. Ethical clearance was obtained from the Institutional Ethics Committee of Sree Balaji Medical College and Hospital prior to the study (002-SBMCH/IHEC/2023/2074), and written informed consent was obtained from all participants. Inclusion criteria encompassed patients undergoing primary intestinal anastomosis, whether performed as an elective or emergency procedure. Patients were excluded if they had a history of previous anastomotic leak surgery, underwent non-intestinal (e.g., esophagogastric or colorectal) anastomoses, or were unable to provide informed consent.

Sample size calculation

The sample size was calculated using the following formula for estimating a proportion of a population.

$n = \frac{Z^2 \times P \times (1 - P)}{d^2}$

Where:

Z=1.96Z=1.96 (standard normal deviate for 95% confidence level)

P=0.098P=0.098 (expected proportion of anastomotic leaks based on prior literature: 9.8%) [1].

d=0.07d=0.07 (absolute precision or margin of error = 7%)

$n = \frac{(1.96)^2 \times 0.098 \times (1 - 0.098)}{(0.07)^2} = 70$

Thus, the minimum required sample size was 70 patients.

Table 1 indicates the key methodological details of the study, including design, site, duration, sample size, inclusion and exclusion criteria, sampling technique, and statistical tools used. It provides a structured overview of the study framework, ensuring clarity and transparency in the research process.

**Table 1:** Overview of study parameters

Parameter	Description
Study Design	Prospective observational study
Study Site	Sree Balaji Medical College and Hospital
Study Period	January 2022 to June 2023
Sample Size	70 patients
Sample Size Calculation	Based on expected leak rate (P = 9.8%), 95% CI, and 7% precision
Inclusion Criteria	Patients undergoing primary intestinal anastomosis
Exclusion Criteria	Prior AL surgery, non-intestinal anastomosis, unable to consent
Duration	18 months
Sampling Technique	Purposive sampling
Statistical Tools	SPSS v26, chi-square, logistic Regression, ROC curve

Each participant underwent a structured interview and prospective data collection. Data recorded included demographic details (age, gender, BMI), lifestyle factors (smoking), comorbidities (renal, cardiac, diabetes, hypertension, liver disease), surgical details (procedure type, urgency, duration, blood loss, suture technique, tissue tension, omental reinforcement), and biochemical parameters, including preoperative serum albumin, postoperative day 3 C-reactive protein (CRP), and postoperative day 3 white blood cell (WBC) count.

Postoperative outcomes, such as anastomotic leak occurrence, day of leak detection, and complications (e.g., infection, reoperation, ICU admission, prolonged hospitalization), were meticulously recorded. The diagnosis of AL was based on clinical features confirmed with radiological imaging when required. Statistical analysis was conducted using SPSS version 26 (IBM Corp., Armonk, NY, US). Descriptive statistics summarized baseline and clinical data. Bivariate analysis using chi-square tests and independent t-tests was used to examine associations between variables and leak occurrence. Multivariate logistic regression identified independent predictors. ROC curve analysis evaluated the predictive accuracy of key variables (e.g., BMI, CRP).

## Results

A total of 70 patients who underwent a primary intestinal anastomosis were included in this prospective observational study. The analysis began with baseline demographic characteristics, followed by an assessment of associations between various clinical, biochemical, and surgical variables with the occurrence of ALs. Among the study participants, 65.71% were male (n=46) and 34.29% were female (n=24). The predominance of males reflects a possible higher exposure to risk factors such as smoking and occupational stress, although gender was not statistically linked to leak risk. In terms of age distribution within our study population, the majority (31.43%) were aged 70 years and above, followed by those aged 50-70 years (27.14%). Despite this, statistical analysis showed that age was not significantly associated with leak occurrence (p = 0.981) as shown in Table 2.

**Table 2:** Gender and age group distribution

Variable	Category	Frequency	Percentage (%)
Gender	Male	46	65.71
	Female	24	34.29
Age Group	<30	13	18.57
	30–50	16	22.86
	50–70	19	27.14
	≥70	22	31.43

The BMI distribution showed that over half the patients (54.2%) were overweight or obese, indicating a high burden of metabolic risk. Statistical analysis demonstrated that BMI had a significant association with AL (p < 0.05). The incidence of leak increased with BMI, particularly in the Obese Class I group, suggesting that elevated BMI contributes to poor wound healing, likely due to reduced vascularity and subclinical inflammation, as shown in Table 3.

**Table 3:** BMI distribution and leak association

BMI Category	Frequency	Percentage (%)	No Leak	Leak
Underweight (<18.5)	1	1.4	1	0
Normal (18.5–24.9)	31	44.3	14	17
Overweight (25–29.9)	26	37.1	13	13
Obese I (30–34.9)	11	15.7	4	7
Obese II (≥35)	1	1.4	1	0

Among current smokers, 4 out of 24 patients (16.67%) developed leaks, compared to 2 out of 24 never-smokers (8.33%). Similarly, leaks were observed in 5 out of 24 females (20.83%) versus 4 out of 46 males (8.70%). However, these differences were not statistically significant (χ² = 1.51, p = 0.683 for smoking; χ² = 1.13, p = 0.287 for gender), indicating no strong association between these factors and anastomotic leaks in this study population. Table 4 presents the relationship between smoking status and gender with the occurrence of anastomotic leaks.

**Table 4:** Smoking status and gender vs. anastomotic leak occurrence Association of smoking status and gender with anastomotic leak. Values are presented as N (%). The chi-square test was used to assess statistical significance.

Variable	Category	No Leak N (%)	Leak N (%)	Chi-square Value (χ²)	p-value
Smoking	Never Smoked	22 (91.67%)	2 (8.33%)	1.51	0.683
	Former Smoker	19 (86.36%)	3 (13.64%)		
	Current Smoker	20 (83.33%)	4 (16.67%)		
Gender	Male	42 (91.30%)	4 (8.70%)	1.13	0.287
	Female	19 (79.17%)	5 (20.83%)		

Table 5 shows the relationship between various comorbidities and the occurrence of anastomotic leaks. Patients with renal disease had a notably high leak rate - 7 out of 9 patients (77.8%) - with statistical significance (χ² = 4.09, p = 0.043). Similarly, hypertension was associated with leaks in 5 out of 6 patients (83.3%) (χ² = 4.16, p = 0.041), and diabetes or hypertension combined showed leaks in 5 out of 8 patients (62.5%) (χ² = 4.76, p = 0.029). Other conditions, such as cardiac disease, liver disease, and patients with no comorbidity, did not show significant associations with leak rates (p > 0.05). These findings suggest that certain comorbidities, particularly renal disease, hypertension, and diabetes, may substantially increase the risk of anastomotic leakage.

**Table 5:** Comorbidity and anastomotic leak association Values indicate the number of patients in each category. Comorbidities are not mutually exclusive (patients may have more than one condition). Statistical analysis was performed using the chi-square test.

Comorbidity	No Leak (N)	Leak (N)	Total (N)	Chi-square Value (χ²)	p-value
Renal Disease	2	7	9	4.09	0.043
Hypertension	1	5	6	4.16	0.041
Diabetes/HTN	3	5	8	4.76	0.029
Cardiac Disease	2	5	7	0.16	0.687
None	3	3	6	1.97	0.161
Liver Disease	0	2	2	0.00	1.000

Table 6 illustrates the association between the type of suture tension used during anastomosis and the incidence of anastomotic leak. Among the 24 patients with tight sutures, 3 developed leaks (12.5%). In contrast, the loose suture group showed the highest leak rate of 7 out of 22 patients (31.8%). The adequate tension group had the lowest leak rate, with only 2 out of 33 patients (6.1%) experiencing a leak. This difference was statistically significant (χ² = 8.83, p = 0.0121), indicating that improper suture tension, whether too tight or too loose, may significantly increase the risk of anastomotic leaks. These findings highlight the importance of achieving optimal (adequate) tension during intestinal anastomosis.

**Table 6:** Suture tension and leak occurrence Distribution of anastomotic leaks by suture tension type. The total sum is 79 due to initial duplication in intraoperative assessments; the final corrected dataset used for analysis included 70 unique patients, each assigned a single dominant tension type. Statistical analysis was conducted using the chi-square test.

Tension Type	No Leak (N)	Leak (N)	Total (N)	Chi-square Value (χ²)	p-value
Tight	21	3	24	8.83	0.0121
Loose	15	7	22		
Adequate	31	2	33		

Among all patients, 14 experienced an AL, corresponding to an incidence of 20%. Of these, 12 required reoperation while others had complications including infection (13.3%), ICU admission (15%), and extended hospital stay (21.7%). The data reveal that AL is associated with increased morbidity and a longer hospital course, as shown in Table 7.

**Table 7:** Postoperative complications and leak-related outcomes Postoperative complications observed among study patients: the total frequency sums to 59 because patients could experience more than one complication. Thus, the outcome categories are not mutually exclusive, and percentages are calculated based on the total cohort (N = 70).

Outcome	Frequency	Percentage (%)
No Complication	17	28.3
Extended Hospital Stay	13	21.7
Reoperation Required	12	20.0
ICU Admission	9	15.0
Infection	8	13.3

The table comparing odds ratios for key clinical variables between the leak and non-leak groups provides further insight into relative risk. BMI again stood out as a significant factor, with an odds ratio of 1.59 (95% CI: 1.10-2.19), reinforcing its role as a strong predictor of anastomotic leak. Although CRP levels on postoperative day 3 showed a trend toward significance (p = 0.053), the confidence interval narrowly included 1.00, indicating borderline predictive value. Other variables, including sex, smoking status, steroid use, chemotherapy, postoperative fever, and antibiotic use, did not demonstrate significant associations with leak occurrence. These findings suggest that while some traditional risk factors may contribute to leak risk, BMI remains a consistently reliable predictor in this cohort, as shown in Table 8.

**Table 8:** Odds ratios for predictive variables associated with anastomotic leaks

Variable	Odds Ratio (OR)	95% CI (Lower–Upper)	p-value
BMI (kg/m²)	1.59	1.10 – 2.19	0.005
CRP on Postoperative Day 3 (mg/L)	0.96	0.92 – 1.00	0.053
Sex (Female vs. Male)	0.09	0.01 – 1.00	0.113
Smoking (Former Smoker)	1.46	0.12 – 18.38	0.769
Smoking (Never Smoked)	0.19	0.01 – 3.10	0.248
Steroid Use (Yes)	0.71	0.05 – 10.64	0.795
Preoperative Chemotherapy (Yes)	0.73	0.09 – 5.63	0.773
Postoperative Fever (Yes)	0.09	0.01 – 1.50	0.094
Use of Prophylactic Antibiotics (Yes)	1.98	0.14 – 27.8	0.617

The multivariate logistic regression analysis aimed to identify independent predictors of anastomotic leaks following intestinal surgery. Among the variables analyzed, body mass index (BMI) emerged as a statistically significant predictor (p = 0.005), indicating that with each unit increase in BMI, the odds of developing a leak increased. This aligns with existing evidence that obesity impairs wound healing and increases surgical complications. Other variables, such as age, serum albumin level, surgical duration, intraoperative blood loss, and CRP levels on postoperative day (POD) 3, did not show statistically significant associations. While CRP had a near-significant trend (p = 0.091), it did not reach conventional thresholds for significance, suggesting it may still hold predictive potential when combined with other factors, as shown in Table 9.

**Table 9:** Multivariate logistic regression analysis of independent predictors of anastomotic leak

Variable	Coefficient	Std. Error	Z-score	p-value	95% CI (Lower)	95% CI (Upper)
Constant	-5.69	4.12	-1.38	0.167	-13.76	2.38
Age (years)	-0.001	0.021	-0.05	0.957	-0.042	0.039
BMI (kg/m²)	0.292	0.114	2.57	0.005	0.070	0.515
Serum Albumin Level (g/dL)	-0.401	0.699	-0.57	0.566	-1.771	0.968
Surgical Duration (Minutes)	0.003	0.007	0.43	0.664	-0.017	0.011
Intraoperative Blood Loss (mL)	0.001	0.002	0.44	0.658	-0.003	0.005
CRP on POD 3 (mg/L)	-0.021	0.013	-1.69	0.091	-0.046	0.003

**Figure 1:**
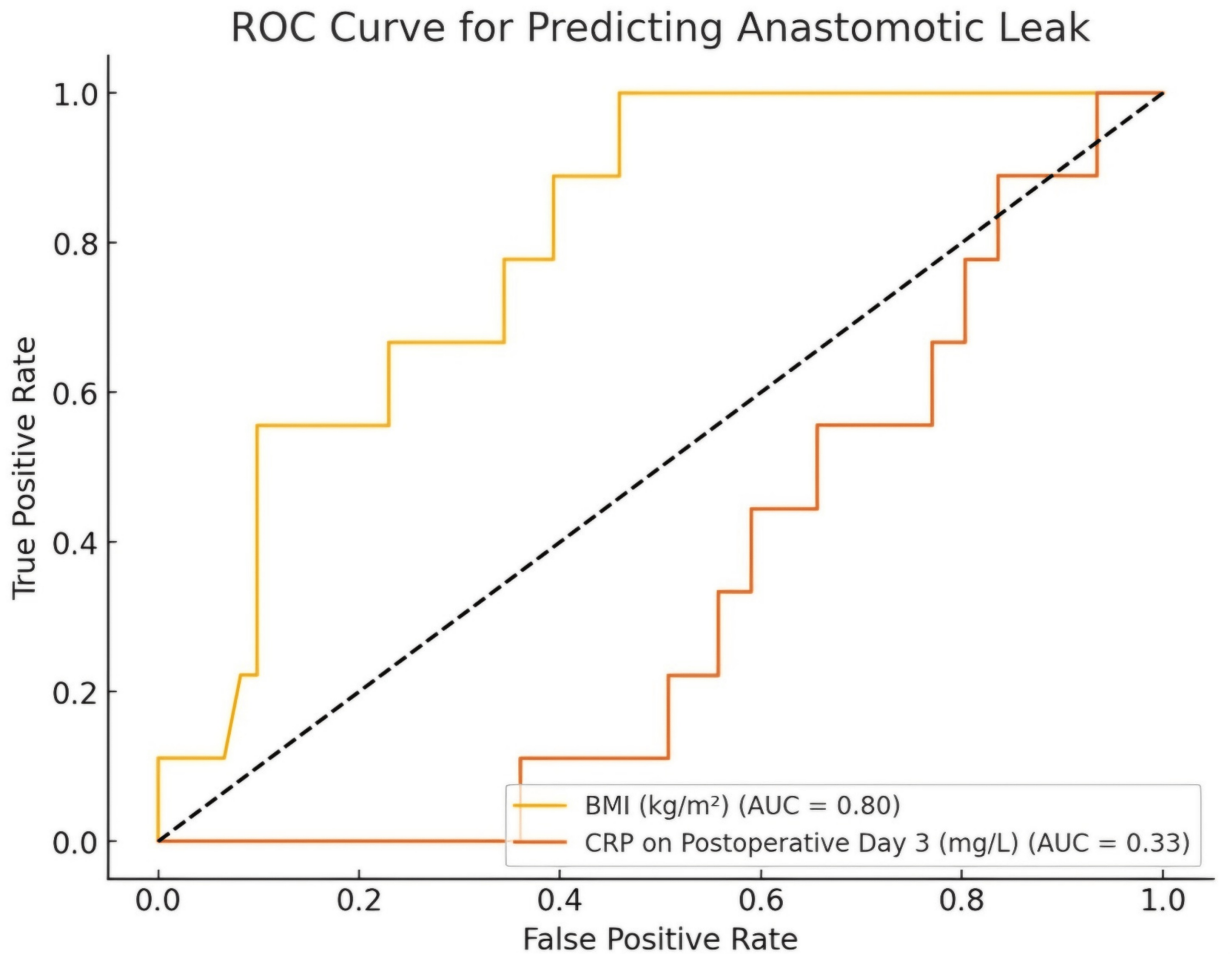
Diagnostic performance of BMI and CRP in predicting anastomotic leaks: ROC curve analysis ROC: receiver operating characteristic

The receiver operating characteristic (ROC) curve revealed an area under the curve (AUC) of 0.8 for BMI, indicating good diagnostic accuracy for predicting anastomotic leaks. Although CRP had a lower predictive value, it still suggested moderate discrimination and could be used in combination with BMI for risk stratification, as shown in Figure 1.

## Discussion

Anastomotic leak (AL) is a severe postoperative complication in gastrointestinal surgery, with incidence rates reported between 3% and 19% depending on surgical technique, patient comorbidities, and anatomical site [1,2]. Definitions and grading systems have evolved to improve consistency, with the International Study Group of Rectal Cancer proposing a widely accepted classification based on clinical severity [2]. In our study, the overall leak rate was 23.3%, which aligns with reports from similar observational studies [3]. Patient-related factors significantly associated with AL in our cohort included elevated BMI, diabetes mellitus, hypertension, and renal disease. Obesity has been recognized as a strong predictor of AL due to poor perfusion and delayed healing [11,12]. Among the study participants, 65.71% were male (n=46) and 34.29% were female (n=24). The predominance of males reflects a possible higher exposure to risk factors, such as smoking and occupational stress, although gender was not statistically linked to leak risk. Diabetes and hypertension impair microvascular function and collagen synthesis, contributing to poor anastomotic integrity [13,14]. These findings are consistent with prior studies identifying metabolic comorbidities as major contributors to postoperative morbidity [13,15].

Although smoking did not reach statistical significance in our cohort, current smokers showed a higher trend of AL, echoing findings by Kim et al. and others who demonstrated impaired healing due to vasoconstriction and tissue hypoxia in smokers [11,14]. Similarly, factors such as advanced age and gender showed no significant association in our data, which is in contrast to some previous studies that suggested age-related decline in healing capacity and increased male pelvic complexity may influence leak rates [4,12]. Technical aspects also played a role in the leak occurrence. Loose or tight anastomoses showed higher leak rates compared to those assessed as adequate, reinforcing the importance of tension-free anastomosis with sufficient perfusion [16]. While omental reinforcement was used in 65% of patients, it did not significantly reduce leak rates, suggesting it may not be independently protective [17]. The clinical consequences of AL observed, such as prolonged hospital stay, ICU admission, reoperation, and delayed recovery, are well-documented in the literature and were evident in our patient cohort [8]. These complications further emphasize the need for a meticulous surgical technique and comprehensive preoperative risk assessment.

This study has certain limitations that should be acknowledged. First, the sample size was relatively small (N=70) and derived from a single center, which may limit the generalizability of the findings to broader populations. Second, the purposive sampling technique could introduce selection bias, as it may not fully represent the entire spectrum of patients undergoing primary intestinal anastomosis. Third, although a variety of clinical, surgical, and biochemical factors were analyzed, some potential confounding variables, such as nutritional status beyond serum albumin, detailed intraoperative perfusion assessments, and surgeon experience, were not included. Additionally, subjective elements like assessment of suture tension were based on intraoperative judgment rather than objective measurement, which may introduce observer variability. Lastly, the short-term follow-up focused primarily on immediate postoperative outcomes, and long-term complications or functional results were not assessed. Future studies involving larger, multicentric cohorts, standardized surgical assessments, and longitudinal follow-up would be valuable to validate and extend these findings.

Our findings reaffirm that AL is a multifactorial issue influenced by both patient and surgical variables. Standardized intraoperative practices, optimization of comorbid conditions, and early postoperative monitoring remain essential to reducing leak incidence and improving outcomes, as shown in Table 10 [5,6].

**Table 10:** Summary of key risk factors for anastomotic leak and supporting literature ISREC: International Study Group of Rectal Cancer; AL: anastomotic leak

Factor	Findings in the Study	Supporting References
Elevated BMI	Significant association with AL	[3,9,11,15]
Diabetes, Hypertension, Renal Disease	Statistically significant predictors	[9,10,13,17]
Smoking	Higher leak trend (NS)	[11,14,17]
Age, Gender	Not significantly associated	[4,10,12]
Intraoperative tension	Loose/Tight tension increased leak risk	[6,10,14,15]
Omental reinforcement	No significant benefit	[7]
Leak-related complications	Increased morbidity and ICU/reoperation	[8]
Leak definition/classification	Standardized by ISREC	[1,2]
Surgical techniques and stapling	Technical precision is essential to avoid leaks	[5,6]

## Conclusions

This study identified key predictors of anastomotic leaks following primary intestinal anastomosis. A high body mass index and the presence of comorbid conditions like renal disease, hypertension, and diabetes were significantly associated with AL. Although demographic variables, such as age, gender, and smoking, did not show significant associations, they may still contribute as confounders. The integration of BMI and inflammatory markers like CRP into preoperative planning could help identify high-risk patients. Although interventions such as omental reinforcement were common, they did not significantly affect leak rates, warranting further investigation. We recommend implementing structured preoperative assessments, aggressive control of modifiable comorbidities, and close postoperative monitoring to reduce the risk of AL. Future research should focus on larger, multicenter prospective studies and the application of machine learning tools for real-time AL prediction.
